# Developmental and lifelong dioxin exposure induces measurable changes in cardiac structure and function in adulthood

**DOI:** 10.1038/s41598-021-89825-w

**Published:** 2021-05-17

**Authors:** Matthew de Gannes, Sheryl E. Koch, Alvaro Puga, Jack Rubinstein

**Affiliations:** 1 Department of Environmental and Public Health Sciences and Center for Environmental Genetics, University of Cincinnati College of Medicine, 160 Panzeca Way, Cincinnati, OH 45267 USA; 2 Department of Internal Medicine, Division of Cardiovascular Health and Disease, University of Cincinnati College of Medicine, 3230 Eden Ave, Cincinnati, OH 45267 USA

**Keywords:** Disease model, Cardiovascular diseases

## Abstract

Congenital heart disease (CHD) is the most common congenital abnormality. A precise etiology for CHD remains elusive, but likely results from interactions between genetic and environmental factors during development, when the heart adapts to physiological and pathophysiological conditions. Further, it has become clearer that early exposure to toxins that do not result in overt CHD may be associated with adverse cardiac outcomes that are not manifested until later life. Previously, interference with endogenous developmental functions of the aryl hydrocarbon receptor (AHR), either by gene ablation or by in utero exposure to 2,3,7,8-tetrachlorodibenzo-*p*-dioxin (TCDD), a potent AHR ligand, was shown to cause structural, molecular and functional cardiac abnormalities and altered heart physiology in mouse embryos. Here, we show that continuous exposure to TCDD from fertilization throughout adulthood caused male mice to underperform at exercise tolerance tests compared to their control and female counterparts, confirming previous observations of a sexually dimorphic phenotype. Renin-angiotensin stimulation by angiotensin II (Ang II) caused measurable increases in blood pressure and left ventricle mass, along with decreased end diastolic volume and preserved ejection fraction. Interestingly, TCDD exposure caused measurable reductions in the myocardial hypertrophic effects of Ang II, suggesting that endogenous AHR signaling present in adulthood may play a role in the pathogenesis of hypertrophy. Overall, the findings reported in this pilot study highlight the complex systems underlying TCDD exposure in the development of cardiac dysfunction in later life.

## Introduction

Congenital heart disease (CHD) is the most common congenital abnormality, accounting for 10–12 cases per 1000 infants and affecting nearly 2 million families in the United States^1–4^. Despite emerging research linking heart development and disease, a comprehensive understanding of the etiology and biological mechanisms for CHD remain elusive. Notwithstanding evidence in human and mouse studies linking mutations in homeobox transcription factor genes governing the early events of heart development with CHD in later life^5–10^, epidemiological studies suggest that a genetic or environmental cause can be identified in 20–30% of CHD cases^11^. Specifically, less than 15% of CHD cases are associated with known Mendelian inheritance^12^, and environmental causes are identifiable in only 2% of cases^13^. Taken together, it can be surmised that a multifactorial etiology arising from a combination of epigenetic, genetic and environmental factors during heart development may be at play^14–16^.

The heart is the first organ to develop during embryogenesis and must adapt to diverse physiological and pathophysiological needs to facilitate organ growth and optimal contractile function^17–19^. Since cardiomyocytes mature with limited cell division after birth, the heart must adapt in new ways to physiological and pathological stressors^20,21^. For example, during adulthood, the cardiac response to a pathologic stressor such as hypertension (increased afterload) is characterized by the development of cardiomyocyte hypertrophy, resulting in associated clinical observations such as left ventricular (LV) hypertrophy (LVH) and subsequent heart failure with preserved ejection fraction (HFpEF)^22^. The development of cardiomyocyte hypertrophy as a precursor to HFpEF is driven by mechanical stress, adrenergic and renin-angiotensin stimulation, and cytokines which activate signaling pathways associated with increased cytosolic calcium and calcium dependence^23^. The myocardial response to a physiologic stressor such as pressure overload due to exercise is associated with alterations in the size and shape of the heart, including LV dilation and eccentric hypertrophy, collectively referred to as ventricular remodeling^24–26^. The above adaptations that occur during heart development may lead to increased risk for heart failure such as HFpEF—now the leading cause of heart failure and related deaths in the United States^27,28^—in the context of pathologic stressors, and sudden cardiac death in the context of physiologic stress^25^.

Exposure to xenobiotic environmental agents that signal through the aryl hydrocarbon receptor (AHR) was shown to disrupt endogenous AHR function and cause adverse cardiovascular effects in several animal and in vitro studies, implicating the AHR in the etiology of cardiovascular disease^29–39^. The AHR is a ligand-activated transcription factor and member of the basic-region helix-loop-helix PER/ARNT/SIM (bHLH-PAS) superfamily^36^. AHR expression can be detected as early as embryonic day (E) E0.5^40^, and by E7.5, AHR expression was detected using immunofluorescence in all three embryonic germ layers (ectoderm, mesoderm, and endoderm) and in the peripheral decidual cells^30^. As an environmental sensor^41^, it is classically known to mediate the metabolism of its xenobiotic ligands via control of the CYP1 family of cytochromeP450s and an array of phase II detoxification enzymes^36^. In recent years, evidence has emerged for important homeostatic and developmental processes regulated by AHR, including immune response, growth factor signaling, cell cycle proliferation, differentiation, arrest, and apoptosis^32,42–44^. A growing body of evidence suggests that the xenobiotic detoxifying role of AHR is an adaptive mechanism that, when exposed to a xenobiotic, derails normal developmental and physiological AHR functions^45^. Indeed, previous work has shown that *Ahr* ablation in mice resulted in a variety of developmental aberrations and pathologies, including neonatal lethality rates, inflammation of bile ducts, depletion of splenic lymphocytes, skin lesions, cardiac hypertrophy, portal vascular hypertrophy, pyloric hyperplasia of the gastrointestinal tract, and patent *ductus venosus*^46–50^. Given the evidence that AHR disruption or ablation of the gene may lead to developmental aberrations and resulting pathologies, it may be conceivable that this may also increase the risk for cardiac pathologies in later life.

TCDD (2,3,7,8-tetrachlorodibenzo-*p*-dioxin; dioxin) is among the most potent AHR agonists^51^ and several animal and human studies have implicated TCDD exposure in the young as being associated with cardiovascular toxicity and pathologies in later life^52–56^. Previous work in fish^52^, birds^53^, and mammals^54^ has shown that the young are more sensitive to TCDD than the adult, and that developmental exposure results in adverse outcomes in adult life. In addition, studies with in utero exposure at E13.5, E15.5, and E18.5 have shown that TCDD results in reduced cardiomyocyte proliferation, altered fetal heart size, disruption of neovascularization, and susceptibility to cardiovascular dysfunction^32,57^. More recently, exposure to TCDD in utero between mouse E7.5 and E11.5 was shown to disrupt endogenous AHR functions and signaling pathways involved in cardiogenesis and cardiac and mitochondrial functions as early as E13.5^39^. Both ten-months old adult *Ahr*^−/−^ and in utero TCDD-exposed *Ahr*^+*/*+^ mice also developed abnormal cardiovascular phenotypes, including hypertrophy, ventricular dilation, increased heart weight, resting heart rate, and systolic and mean blood pressure (BP), and decreased exercise tolerance^38^. Finally, activation of the AHR/TCDD axis in AHR-positive differentiating ES cells disrupted the expression of genes that regulated signaling pathways related to cardiac morphogenesis and differentiation and implicated in pathologies such as cardiac hypertrophy and cardiac arrhythmias^30^.

Taken together, these studies suggest that both in vitro and in vivo disruption of AHR homeostatic and developmental functions by TCDD impairs postnatal cardiovascular maturation, structure, and function, and this may have the potential to cause cardiac insufficiency associated with congenital heart disease in humans. Notwithstanding the aforementioned evidence, we sought to expand the above body of data in light of recent observations that AHR can be detected as early as E0.5^40^ and because TCDD induces the formation of CYP1A4 in cardiomyocytes and in perivascular cells^58^ which are located in the same region as cardiac impulse-conducting Purkinje cells^59^. In addition, the much longer half-life of TCDD in humans (7–10 years) than in rodents (10–30 days)^60^ warrants new investigations to elucidate the consequences of continuous TCDD exposure throughout life. Thus, to test the hypothesis that TCDD exposure during conception, fetal life, infancy, and early adulthood predisposes it to altered cardiac structure and function associated with cardiac disease, we implemented a pilot investigation to study male and female mice that were exposed to TCDD from conception (E-0.5) through adulthood up to 14 months of age. Changes in cardiac structure and function were assessed at E15.5 by echocardiography, at post-natal day (PND) 5 by echocardiography and EKG, and from 2 months of age to 14 months every other month by echocardiography and EKG. To evaluate the effects of physiological stressors such as exercise, tests for exercise tolerance were also performed at 12 months of age. Finally, to evaluate the structural and functional cardiac effects of pathologic stress induced by angiotensin II (Ang II) in TCDD-treated 14-month-old mice, BP, echocardiography, and immunohistochemistry studies were performed after 2 weeks of angiotensin II-induced pathologic stress.

## Materials and methods

### Animals and treatments

**Figure 1:**
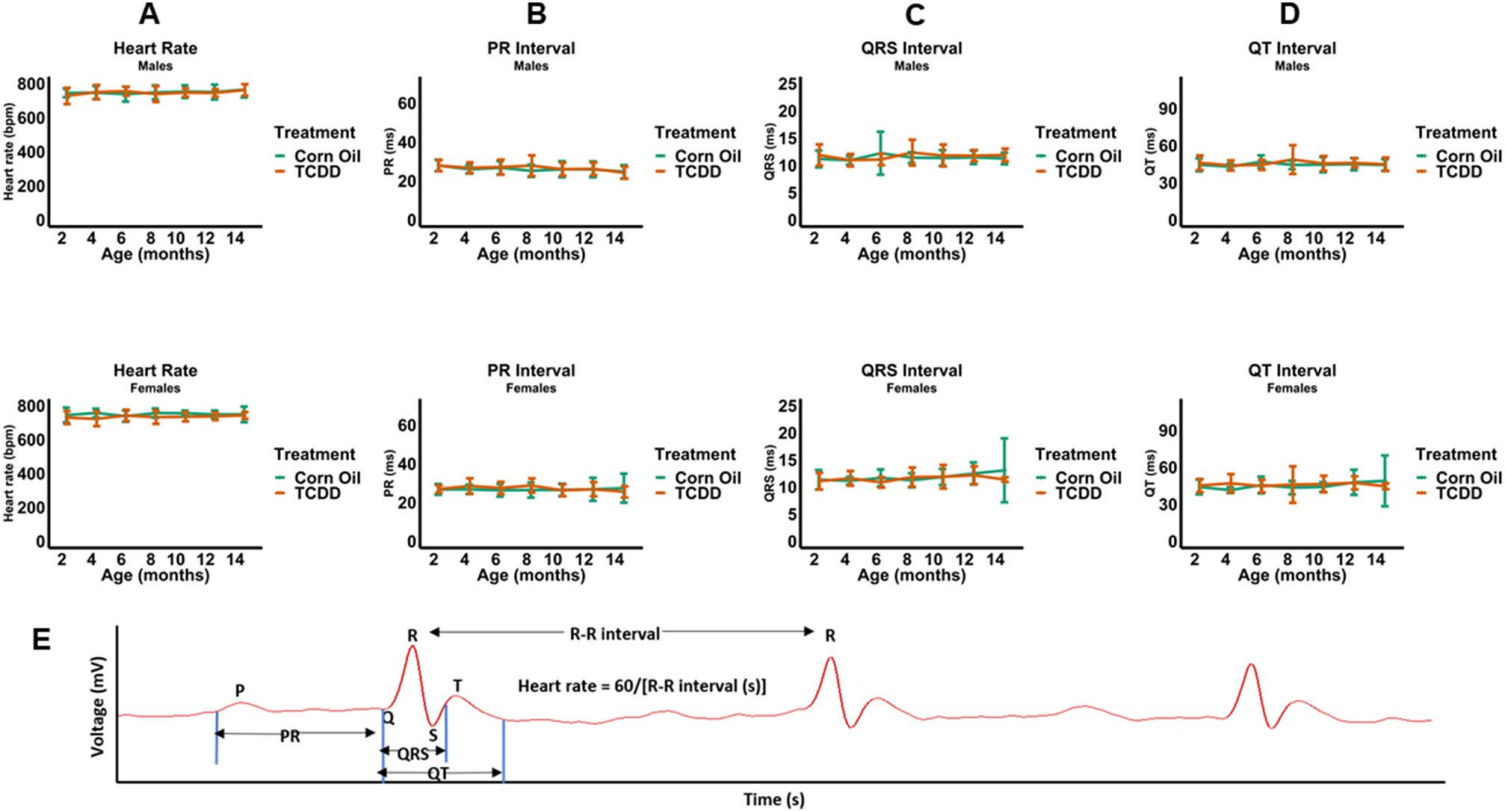
Effect of TCDD on the electrical function of male and female murine hearts over 14 months continuous exposure. Heart rate (**A**) and the electrical parameters PR (**B**), QRS (**C**), and QT (**D**) intervals were assessed. A representative annotated EKG plot from one of the above experiments (**E**) is illustrated. All data are expressed as mean + SD.

All experiments were conducted using the highest standards of humane care in accordance with the NIH Guide for the Care and Use of Laboratory Animals and were approved by the University of Cincinnati Institutional Animal Care and Use Committee (IACUC). The approved IACUC numbers are 12-09-18-01 and 11-02-22-01. C57BL/6J mice were initially obtained from The Jackson Laboratory in Bar Harbor, ME. Female mice were mated overnight with male mice. Maternal gestational exposure to the prototypical AHR ligand TCDD was performed via oral gavage starting at key developmental time points as previously described^30,38,39^ and is illustrated in detail in Fig. 1 in Online Resource 1. Dams were treated by oral gavage on embryo days E-0.5 (the day of mating), E7.5, and post-natal day (PND) 10 with either corn oil (vehicle) or with TCDD at a dose of 1 µg/kg, which based on previous determinations is estimated to correspond to 0.34 ng per embryo^61^, and has been previously shown to affect a variety of genes regulated by AHR signaling^38,39^. Offspring of dams were weaned at PND21 and males and females were kept separately in standard housing conditions until the scheduled necropsy after 14 months of age. All offspring were divided into cages according to their mothers’ treatment, and treated with either corn oil or TCDD (same dose used for dams) every week starting from weaning until 14 months of age. TCDD doses were specifically selected to include environmentally relevant ranges and are within the range of reported body burdens of populations with known exposure for dioxin and dioxin-like compounds, in the range of 0.1–7 µg TEQ/kg^62^. The health of all animals receiving treatment by oral gavage was monitored daily veterinary staff for any signs of distress, irregular breathing, malnutrition, or harmful physical manifestations. Mice were euthanized at the end of the study using compressed carbon dioxide followed by cervical dislocation per the University of Cincinnati’s IACUC protocols. All surgery was performed using isoflurane anaesthesia, and all efforts were made to minimize suffering. Mortality occurring outside of planned euthanasia was identified by the university’s veterinary staff and did not differ between TCDD-treated and control mice. All experiments meet the guidelines outlined in the ARRIVE Guidelines 2.0 authors’ checklist (Online Resource 2) to ensure reliability of methods and findings.

### Osmotic minipump implantation surgery

At 14 months of age, osmotic minipumps (model 2002, Alzet, Cupertino, California, USA) containing either Ang II (1.44 mg/kg/day) or saline (0.9% sodium chloride; B. Braun Medical Inc, Irvine, California, USA) were implanted subcutaneously into mice under anesthesia with isoflurane 2.5%/oxygen mixture at 2 l/min for pump installation. The pumps were kept in the mice at a flow rate of 0.5 µl/h for 14 days before BP and echocardiography measurements and necropsy were performed.

### Echocardiography and electrocardiography

**Figure 2:**
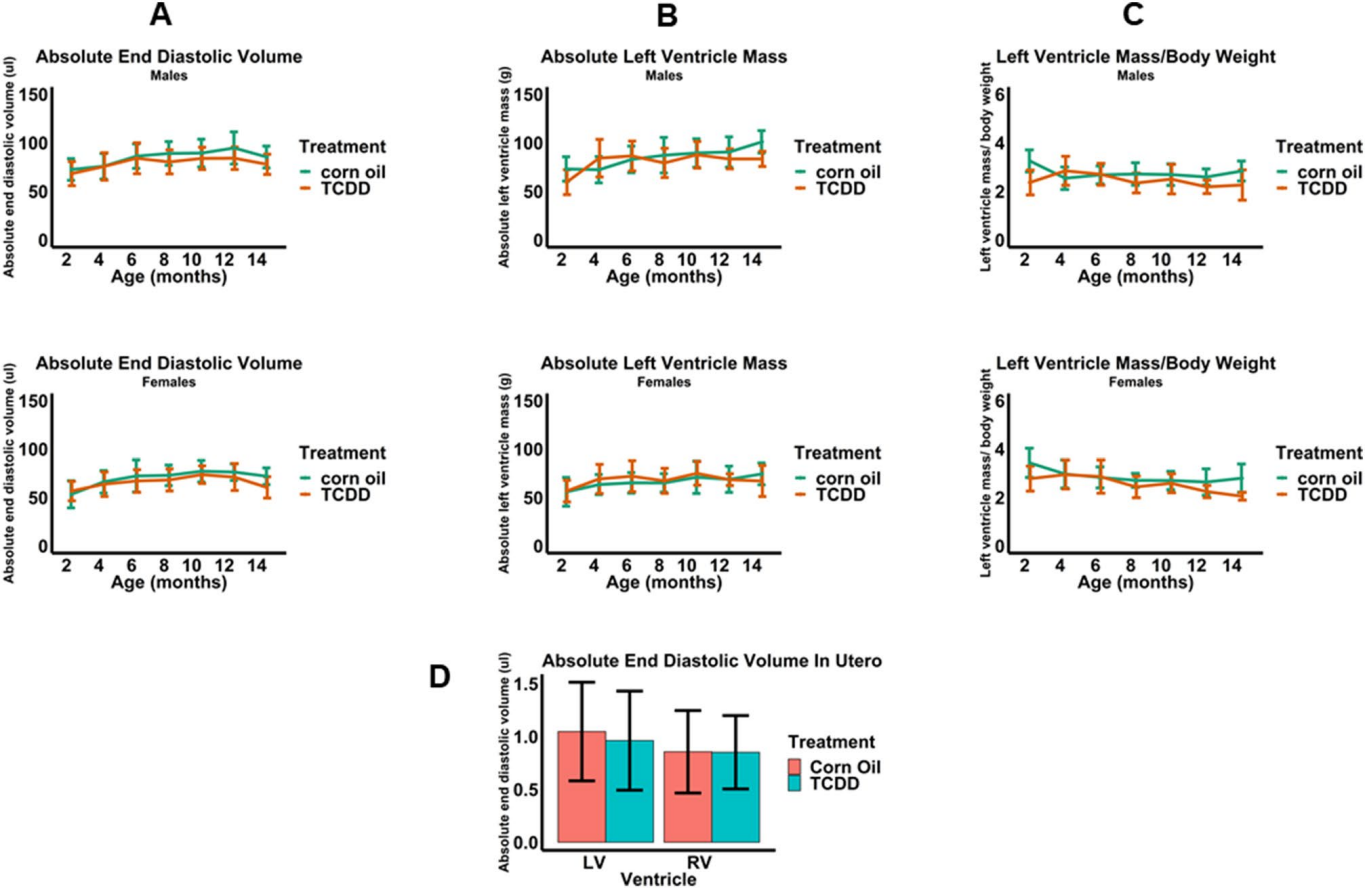
Echocardiographic assessment of cardiac structure after continuous exposure to TCDD for 14 months. End diastolic volume (**A**), absolute left ventricle mass (**B**), and left ventricle mass normalized to body weight (**C**) were assessed in males and females after 14 months of continuous exposure to TCDD. End diastolic volume was assessed after exposure to TCDD in utero at E15.5 (**D**). All data are expressed as mean + SD.

**Figure 3:**
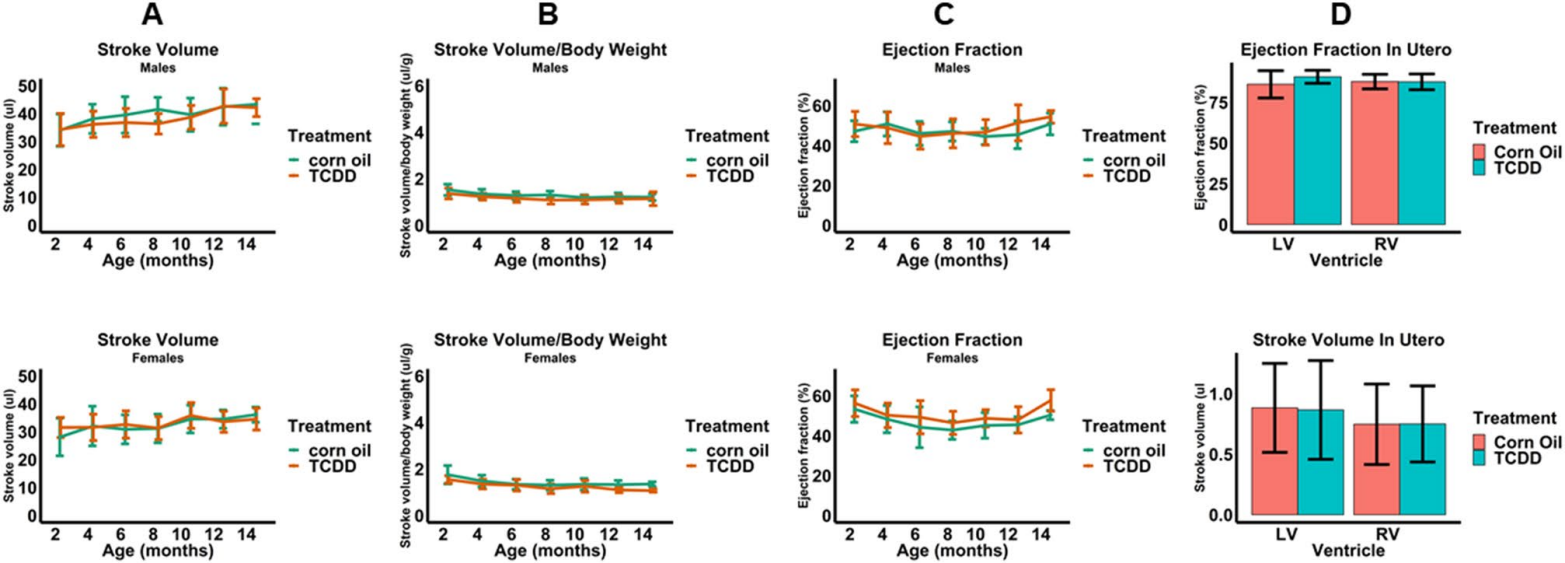
Echocardiographic assessment of cardiac function after continuous exposure to TCDD for 14 months. Stroke volume (**A**), stroke volume normalized to body weight (**B**) and ejection fraction (**C**) were assessed in males and females after 14 months of continuous exposure to TCDD. Ejection fraction and stroke volume were assessed after exposure to TCDD in utero at E15.5 (**D**). All data are expressed as mean + SD.

To determine structural and functional heart abnormalities, high-frequency echocardiographic studies were carried out in utero at E15.5 and every other month from 2 to 14 months of age as previously described^63,64^. Postnatal mice were anesthetized with isoflurane (2.5%), and images were obtained from a parasternal long axis view between 2 and 10 mm in depth in both M-mode and B-mode. Images were taken using the Vevo 2100 Ultrasound system equipped with a MS400 18–38 MHz transducer and postprocessed (Visualsonic, Vevo 2100, v1.1.1 B1455). An illustrative example of these images is shown in Figs. 2 and 3 in Online Resource 1. M-mode images were post-processed for cardiac functional analysis including ventricular size, ejection fraction and cardiac output. At 14 months of age, echocardiography studies were carried out at baseline and two weeks after osmotic minipump implantation surgery. All mouse sample sizes for these studies are shown in Table 1 in Online Resource 1.

Electrocardiography (EKG) measurements were taken noninvasively without anesthesia with an EC Genie (Mouse Specifics, Framingham, MA) as described previously^65^ and at specific time points as illustrated in detail in Fig. 1 in Online Resource 1. Briefly, animals were placed on a gel electrode lead plate on the EKG recording platform. After 4–10 min of acclimation, EKG signals were recorded for 5–10 min until there were at least 30 s of consistent EKG signals free of movement artefacts. Raw EKG data were signals averaged across successive R-R intervals. When necessary, a cotton swab was used to gently move the animal’s feet into a proper position on the lead plate for recording. All mouse sample sizes for these studies are shown in Table 1 in Online Resource 1.

### Blood pressure

At 14 months of age, mice were subjected to noninvasive tail cuff [(Kent Scientific, CODA Standard, (Kent Scientific Corporation, Torrington, Connecticut, USA)] BP measurements as indicated by manufacturer guidelines. Measurements were taken at baseline and two weeks after osmotic minipump implantation surgery. Twenty cycles of measurements were performed. Systolic BP and mean arterial pressure (MAP) were determined.

### Histopathology

Histopathology was performed as previously described^66^. Briefly, at 14 months of age and two weeks after osmotic pump implantation surgery, hearts were formalin-fixed, routinely processed, paraffin embedded, and cut into 6-µm sections, and individually stained with Masson’s Trichrome (MT; Masson Trichrome Stain Kit, ThermoScientific, Kalamazoo, MI). Ten non-overlapping photomicrographs were taken for each mouse (Olympus 1X71; Olympus, Melville, New York) and then randomized before analysis. Quantification of MT-stained sections was performed via color based thresholding assisted by ImageJ (NIH) as previously described^67^.

### Exercise tolerance

Environmental cardiovascular challenge was performed by using an Accupacer Treadmill (Omnitech Electronics, Inc) and assessed at 12 months of age, adapted from previously described methods^68,69^. The mice were subjected to three different treadmill protocols spread over four consecutive days. First, to acclimate the mice to the equipment, they were run for two days following the protocol in Table 2 in Online Resource 1. Any mice that did not respond to shocks or failed to run were excluded from the analyses. Second, on the third day, the mice were given a physical capacity test to verify the speed at which they are capable of running. For the physical capacity test, the mice first followed the protocol in Table 2 in Online Resource 1, then the treadmill was set to 14 m/min and the shock strength was set to 2.0 mA. The speed increased by 2 m/min every 20 s until failure. Lastly, on the fourth day, the mice were subjected to endurance testing. Following the protocol in Table 2 in Online Resource 1, the mice were run at 14 m/min for 60 min and the shock strength was set to 2.0 mA. The time of each mouse’s failure was recorded. Shocks were only counted when one of the following criteria were met: the mouse fell onto the shock bars, any part of the mouse touched the metal shock bars (exception: the mouse’s tail) or the mouse noticeably jumped from the shock. Failure was defined as the mouse can no longer run, the mouse does not respond to a shock or the mouse has reached a total of 50 shocks for the day. After failure, a mouse was immediately returned to its cage.

### Statistical analysis

All statistical analyses was performed using R version 4.0.0. Significant changes due to treatment and sex in echocardiography, EKG, histology quantifications of cardiac fibrosis and BP, measurements were assessed by unbalanced two-way analysis of variance (ANOVA) with a significance threshold of *p* ≤ 0.05. For exercise tolerances measurements, significant interaction between treatment and sex were assessed using non-parametric two-way analysis of covariance (ANCOVA) and significant changes were assessed separately for males and females using two-sided Mann–Whitney U test. Males and females were also assessed separately for Ang II and TCDD effects by sex in echocardiography, histology, and blood pressure measurements using Welch two-sided t-test. For echocardiography and EKG measurements performed for the same mice under different time points, repeated-measures two-way ANOVA was performed.

## Results

### TCDD exposure and angiotensin did not impact body weight

**Figure 4:**
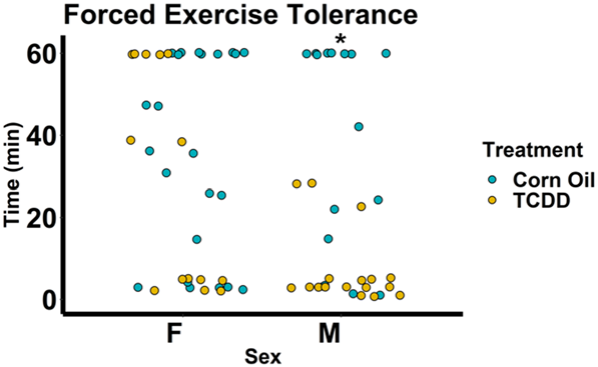
Forced exercise tolerance after TCDD exposure in mice from E0.5 through 12 months of age. The mice were run at 14 m/min for 60 min and the shock strength was set to 2.0 mA. Shocks were only counted when one of the following criteria were met: the mouse fell onto the shock bars, any part of the mouse touched the metal shock bars (exception: the mouse’s tail) or the mouse noticeably jumped from the shock. Failure was defined as the mouse can no longer run, the mouse does not respond to a shock or the mouse has reached a total of 50 shocks for the day. After failure, a mouse was immediately returned to its cage. Each dot represents an individual mouse. *p < 0.05 as measured by two-sided Mann–Whitney U test for a specific sex. Corn oil: n = 23 females, 16 males; TCDD: n = 14 females, 17 males. One male mouse was excluded due to inability to respond to shocks during acclimatization.

**Figure 5:**
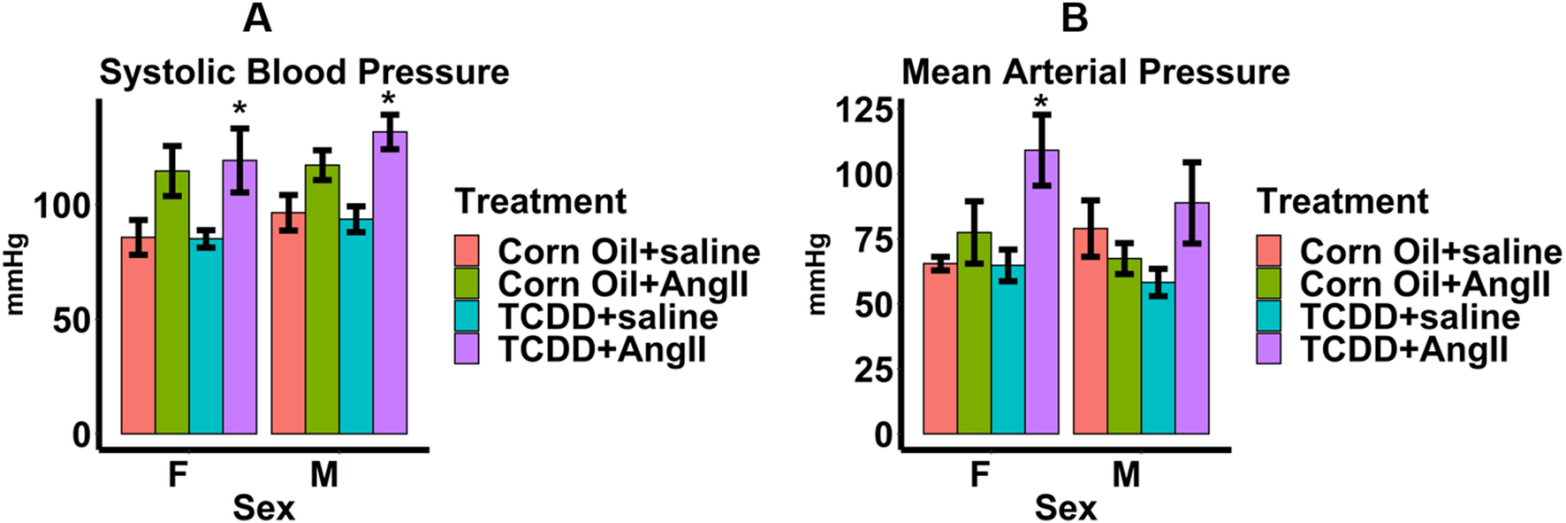
Blood pressure measurements taken two weeks after angiotensin II infusion in male and female 14-month-old mice. Systolic blood pressure (**A**) and mean arterial pressure (**B**) were measured. Values are mean + SE. *p < 0.05 using Welch two-sided t-test comparing saline to Ang II by each sex and either corn oil or TCDD. ^#^*p* < 0.05 using Welch two-sided t-test comparing TCDD to corn oil by each sex and either saline or Ang II. Corn oil + Ang II: n = 6 females, 7 males; Corn oil + saline: n = 4 females, 4 males; TCDD + Ang II: n = 7 females, 7 males; TCDD + saline: n = 6 females, 7 males.

**Figure 6:**
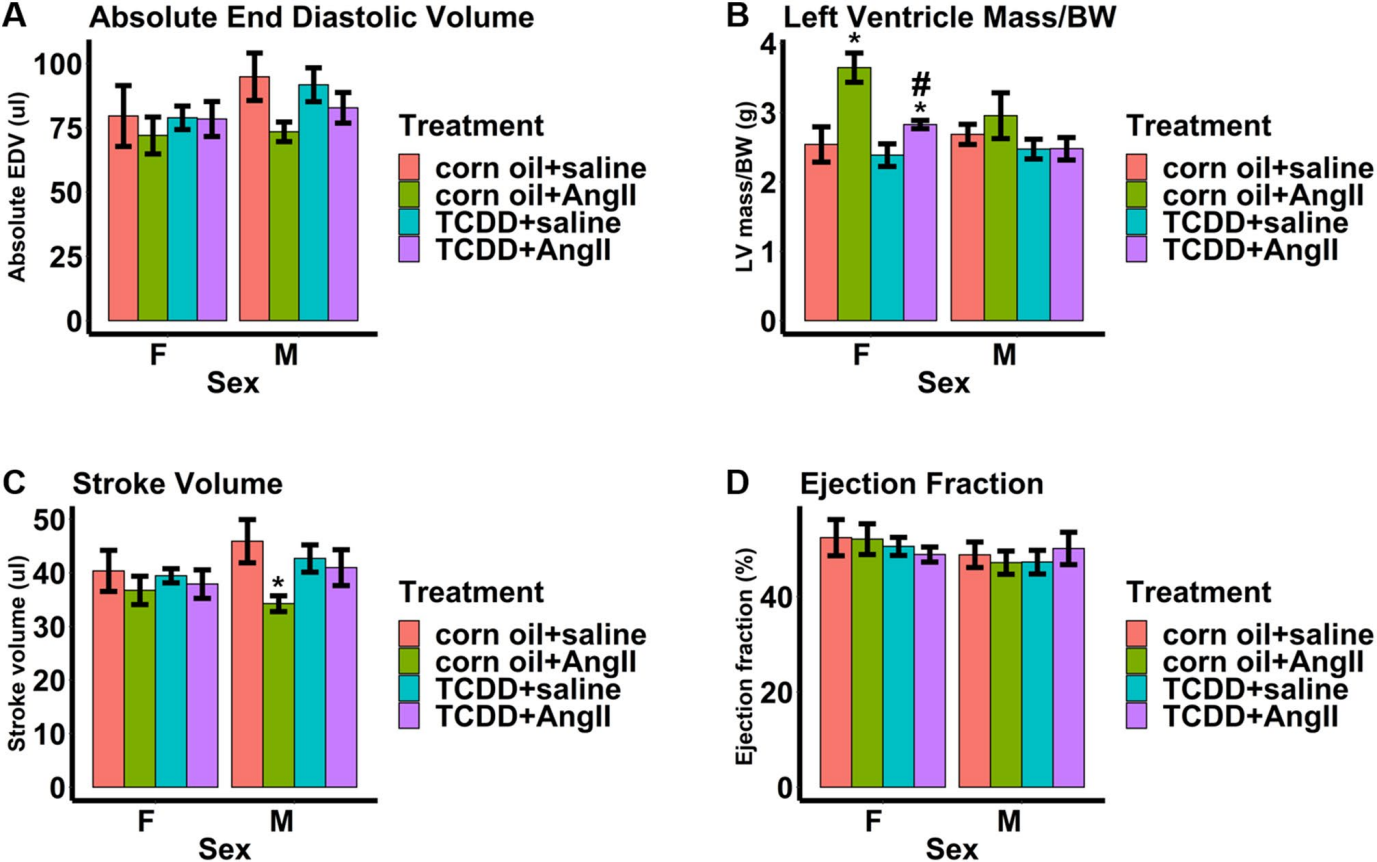
Echocardiography measurements of cardiac structure for mice with osmotic pump implants. Absolute end diastolic volume (**A**), left ventricle mass/BW (**B**), stroke volume (**C**), and ejection fraction (**D**). Values are mean + SE. *p < 0.05 using Welch two-sided t-test comparing saline to Ang II by each sex and either corn oil or TCDD. ^#^*p* < 0.05 using Welch two-sided t-test comparing TCDD to corn oil by each sex and either saline or Ang II. Corn oil + Ang II: n = 6 females, 7 males; Corn oil + saline: n = 4 females, 5 males; TCDD + Ang II: n = 7 females, 7 males; TCDD + saline: n = 6 females, 9 males. *BW* body weight, *EDV* end diastolic volume, *LV* left ventricle.

For the 14 month duration of the study, there was no statistically significant difference in body weight between TCDD-treated mice and controls among both males and females (Figs. 4 and 5 in Online Resource 1). In addition, there was also no statistically significant difference in body weight between TCDD-treated mice and controls, regardless of whether mice were implanted with Ang II or saline (Fig. 6 in Online Resource 1).

### Early developmental TCDD exposure impaired exercise endurance capacity in male mice

Previously, developmental exposure to TCDD and disruption of AHR was found to affect adult cardiovascular structure and function^38^. We aimed to extend these observations based on recent findings that *Ahr* expression can be detected as early as E0.5 after fertilization^40^ by exposing mice in utero starting at this stage of development, and continuing TCDD exposure from birth through adulthood to recapitulate continuous TCDD exposure in adult humans with longer half-lives. Testing the hypothesis that disruption of AHR by TCDD from fertilization through adulthood results in long-lasting consequences to heart structure and function in adult mice, we performed EKG studies examining heart electrical function, echocardiographic studies examining heart structure and function, exercise endurance capacity after forced treadmill exercise, and studies assessing the structural and functional effects of Ang II pathological stress.

EKG assessments showed that, compared to corn oil (control) mice, continuous TCDD exposure did not affect heart rate, or PR, QRS, and QT intervals (Fig. 1A–D). A representative EKG plot from one of the experiments is also illustrated in Fig. 1E. Echocardiographic assessments of cardiac structure revealed that, relative to control, neither males nor females had significant alterations in end diastolic volume (EDV), absolute LV mass, or LV mass normalized to body weight (Fig. 2A–C). Echocardiographic assessments in utero at E15.5 also showed no alterations in EDV (Fig. 2D). Finally, echocardiographic assessments of cardiac function showed no effects of TCDD on stroke volume, stroke volume normalized to body weight, and ejection fraction in males nor females (Fig. 3A–C) nor on ejection fraction and stroke volume in utero (Fig. 3D).

Exercise endurance, measured by the time lapsed on a forced treadmill exercise protocol until failure, differed significantly between males and females among TCDD-treated 12-month-old mice (Fig. 4). Specifically, among females, TCDD had no effect on exercise endurance, but among males, those treated with TCDD significantly underperformed, lasting 50–95% less than their control counterparts. There was also a significant interaction between treatment and sex on exercise tolerance.

### TCDD impacted cardiac structure and function after angiotensin-induced pathological stress

**Figure 7:**
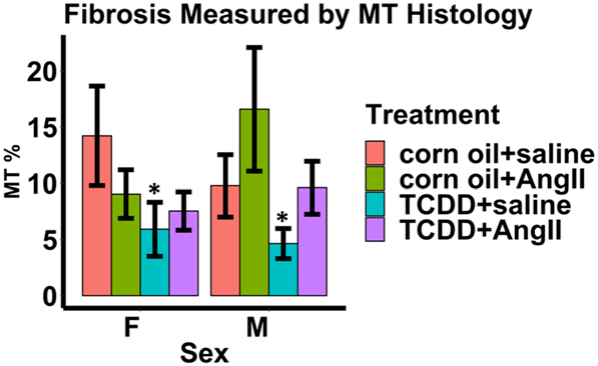
Fibrosis measured by Masson’s Trichrome (MT) staining in hearts obtained from 14-month old mice. Two weeks after osmotic pump implantation surgery, hearts were formalin-fixed, routinely processed, paraffin embedded, and cut into 6-µm sections, and individually stained MT. Ten non-overlapping photomicrographs were taken for each mouse and then randomized before analysis. MT-positive quantification was performed via color based thresholding assisted by ImageJ (NIH) to determine the percentage of MT positive tissue indicative of fibrosis (% positive index). Values are mean + SE. *p < 0.05 using Welch two-sided t-test comparing saline to Ang II by each sex and either corn oil or TCDD. ^#^*p* < 0.05 using Welch two-sided t-test comparing TCDD to corn oil by each sex and either saline or Ang II. Corn oil + Ang II: n = 6 females, 7 males; Corn oil + saline: n = 4 females, 4 males; TCDD + Ang II: n = 7 females, 7 males; TCDD + saline: n = 6 females, 7 males.

To verify the effect of continuous TCDD exposure on cardiac structure and function, we subjected male and female control and TCDD-treated mice to 2 weeks of continuous infusion of Ang II or saline. To validate Ang II treatment, we measured whether there were expected increases in blood pressure in both corn oil and TCDD-treated male and female mice infused with Ang II compared to saline (Fig. 5A,B).Significant increases in systolic blood pressure due to Ang II were observed only among TCDD-treated male and female mice. There were trends towards increased systolic blood pressure due to Ang II among corn oil-treated male and female mice, though these were not significant. Similarly, mean arterial pressure was also increased among TCDD-treated female mice receiving Ang II. There was a trend towards increased mean arterial pressure due to Ang II among male mice, though this was not significant. No significant changes in mean arterial pressure due to Ang II were observed among corn oil-treated mice. When assessing the effect of Ang II on cardiac structure through echocardiography, there was a trend towards Ang II-induced reduced absolute EDV in both male and female corn oil-treated mice compared to saline groups, although these changes were not significant (Fig. 6A). Interestingly, TCDD appeared to mitigate these changes in both sexes, with absolute EDV levels close to those found in saline groups, but these changes were also not significant. Similarly, Ang II caused increases in LV mass normalized to body weight in both sexes among corn oil groups compared to those treated with saline, although these changes were only significant among females (Fig. 6B). TCDD also appeared to mitigate this effect, with LV masses closer to those found in saline groups in both sexes and a significant reduction among female mice. When assessing the effect of Ang II on cardiac function, Ang II reduced stroke volume in males compared to saline groups (Fig. 6C). TCDD also appeared to mitigate this effect, with stroke volume levels approaching those found in saline groups, although this change was not significant. There were no significant effects of Ang II or TCDD on stroke volume in female mice Neither Ang II nor TCDD appeared to have any effects on ejection fraction among both sexes (Fig. 6D). Interestingly, TCDD appeared to also significantly reduce fibrosis among saline-treated male and female mice (Fig. 7).In addition, TCDD caused a trend towards reduction of fibrosis towards saline levels among Ang II-treated male mice but this trend was not significant. Taken together, the data indicate measurable evidence that the effects of Ang II on cardiac structure and function may be mitigated by TCDD.

## Discussion

To extend previous observations that disruption of the Ah receptor signaling network by TCDD in utero causes abnormal cardiac structure and function^38^, this study expanded TCDD treatment to include E0.5 (fertilization, when *Ahr* is first detected^40^), neonatal, and adult weekly exposures to TCDD. We find that early developmental and continued exposure to TCDD did not affect the electrical, structural or mechanical functions of the heart on its own, but dramatically reduced exercise tolerance in male mice. Interestingly, continuous exposure to TCDD also showed measurable trends towards reduction of pathological effects of Ang II on cardiac structure and function, suggesting a potential role for AHR signaling in conferring resistance to hypertrophic development. The trends were not significant, but future studies employing larger sample sizes with greater statistical power and capabilities may serve to validate the above findings.

We investigated the effect of TCDD on the heart’s electrical function because disruption of the TCDD/AHR axis was found to implicate genes and pathways involved in cardiac arrhythmias^30^. In addition, in situ hybridization and immunohistochemical studies showed that, in the chick embryo heart, TCDD induces the formation of CYP1A4 in cardiomyocytes and perivascular cells^58^ which are located near impulse-conducting Purkinje cells^59^. Our EKG studies demonstrated that early and continued TCDD exposure through 14 months did not affect heart rate, nor the PR, QRS, or QT intervals, suggesting that TCDD treatment does not affect sinus node function, conduction, depolarization, or repolarization of the ventricles. These results represent the first study investigating consequences to electrical rhythm in mice as a result of TCDD exposure. To date, studies involving other animal models have been mixed. One study involving chick embryos showed that *in ovo* exposure to TCDD dissolved in dioxane at 1 or 2 nmol per egg for 48 h in 16–18 day old chick embryos before performing ECG measurements produced no effect on PR, QRS, or QT intervals^70^. On the other hand, *in ovo* administration of 0.24 or 0.3 pmol/g TCDD on incubation day 0 increased the incidence of arrhythmias in chick embryos on incubation day 10^71^. In rats aged between 3 and 4 months old, treated weekly with 2 µg/kg/week TCDD in corn oil for 45 days, TCDD exposure led to an elevation in the PR, QRS, and QT intervals compared to the control group. Taken together with our results, the data to date suggest that further studies investigating the consequences to cardiac rhythm of TCDD exposure are warranted in mice, especially at developmental time points and treatment intervals previously used to assess disruption of AHR by TCDD.

Building on previous evidence indicating that disruption of AHR by in utero exposure to TCDD causes abnormal cardiac structure and function in adult mice^38^, we performed further echocardiography studies examining the consequences of continuous TCDD exposure throughout life, starting at fertilization (E0.5). Using the same high-dose, 1 µg/kg TCDD treatment protocol as previously described^38^, we found that early TCDD exposure did not affect EDV, ejection fraction, or stroke volume in utero at E15.5, nor EDV, ejection fraction, stroke volume or LV mass (even normalized to body weight) after continuous exposure through 14 months. Previously, TCDD was administered only at E7.5, E9.5, and E11.5 which resulted in significant decreases in LV mass (both absolute and normalized to body weight) in adults, but no changes in ejection fraction or stroke volume^38,39^. Interestingly, ablation of the *Ahr* gene was associated with higher stroke volumes and reduced ejection fraction relative to naïve hearts in one of these studies, suggesting that disruption by TCDD alone in this context is not enough to overcome compensating homeostatic mechanisms^39^. Other studies demonstrated significant increases in cardiac hypertrophy due to exposure to TCDD both in utero^44^ and during lactation^72^, but the doses utilized were several fold higher than the dose used in this study, which was specifically selected to be within the environmentally-relevant range and reported body burdens of populations with known exposure for dioxin and dioxin-like compounds^62^.In addition, cardiac hypertrophy was also found in adult male mice exposed to TCDD by oral gavage three times per week for 60 days^73^, suggesting that sustained AHR activation during adulthood, in the absence of compensation mechanisms that may arise through exposure during development, may lead to adverse cardiac outcomes. Notwithstanding the possibility that AHR disruption by TCDD during fertilization may be inconsequential to early heart development, the discrepancy between results for cardiac hypertrophy suggests a critical window of susceptibility during which targeted TCDD exposure may likely impact heart developmental steps that affect LV mass. Apart from TCDD exposure at E0.5, we performed in utero TCDD treatment only at E7.5, during which time the induction of cardiogenic mesoderm from splanchnic mesoderm and the critical initiation of events in commitment toward cardiomyogenesis occur^74^. Since the formation of ventricles from the elongated and looped heart tube occurs between E9.5 and E11.5, with septation and remodeling completing around E14.5, it is possible that TCDD treatment during this period has a stronger bearing on LV mass than at earlier time points of development. In addition, mice in our study were also exposed to TCDD via oral gavage weekly from weaning until 14 months of age, raising the possibility of potential regulatory checkpoints to suppress AHR activity as a result of continuous TCDD exposure. The AHR repressor (AHRR) is activated by the liganded AHR complex and inhibits AHR function by competing with AHR for dimerizing with AHR nuclear translocator and binding to the xenobiotic response element to induce its effects^75^. Notwithstanding emerging evidence that AHRR may serve a protective role against TCDD-induced pathologies such as tumor growth and inflammation^76,77^, very little is still known about its function in regulating AHR in different contexts. Future studies that measure both AHR and AHRR activity in relation to the TCDD exposure regimen and phenotypes observed in this study may provide further mechanistic evidence for a potential regulatory role against AHR in the context of continuous TCDD exposure. Taken together, the results from our study and others add further credence to the notion that the consequences of disruption to the AHR/TCDD axis are specific to the developmental and spatial patterns of AHR expression, and that homeostatic mechanisms in certain cells and developmental time points may compensate for the disruption of AHR.

Similar to recent findings^39^, despite the absence of alterations to cardiac structure and function measured by echocardiography, male mice exposed to TCDD displayed decreased exercise tolerance under strenuous forced running. Our results captured over 14 months of continuous TCDD exposure were even more dramatic than those reported previously over 9 months, with males significantly underperforming compared to their control counterparts by up to 95% less time elapsed. TCDD treatment did not affect exercise tolerance in female mice, suggesting a sexually dimorphic effect. Exercise is a complex physiological stressor that is a function of optimal performance of the heart myocardium, made up of cardiomyocytes, to deliver energy and oxygen requirements to cells^78^. Notwithstanding other potential factors that affect exercise tolerance such as learning that may be influenced by TCDD^79^. Mitochondria are crucial for cardiomyocyte survival and function and make up 20–30% of the cell volume of cardiomyocytes. These numbers increase with enhanced myocardial energy requirements such as exercise. Among the many features of aging, a phenomenon that may be exacerbated by TCDD^80^, mitochondrial dysfunction can occur as a result of continuous exposure to both exogenous and endogenous threats, compromising nuclear and mitochondrial DNA integrity^81,82^. Since mitochondrial capacity is highly correlated with positive exercise outcomes^83^, it would appear that our results, building upon those found previously^39^, suggest that AHR disruption by TCDD impairs exercise tolerance further with age via mechanisms that may include cardiovascular performance and energy metabolism. In addition, sexually dimorphic differences in the expression of mitochondria-related genes have previously been reported^84^, which may explain the differential response to exercise between males and females in our study. Taken together with previous epidemiological reports of sex-dependent dimorphic differences in the prevalence of cardiac disease^12,85^, our results suggest that age and sex may be important risk factors for TCDD-induced poor exercise performance.

The cardiac response to a pathologic stressor such as hypertension (increased afterload) is characterized by the development of cardiomyocyte hypertrophy, resulting in associated clinical observations such as LV hypertrophy (LVH) and subsequent heart failure with preserved ejection fraction (HFpEF). HFpEF is a condition in which the muscles of the heart contract normally, pumping a normal proportion of blood, but the LV fails to fill, therefore holding too small a volume of blood to meet the body’s requirements^22^. The development of cardiomyocyte hypertrophy as a precursor to HFpEF is driven by mechanical stress, adrenergic and renin-angiotensin stimulation, and cytokines which activate signaling pathways associated with increased cytosolic calcium and calcium dependence^23^. These factors influence the progression of LVH from a ‘compensated’ state to ‘heart failure’ with preservation of LV systolic function but impaired LV filling. We sought to determine whether our echocardiographic findings that TCDD had no effect on cardiac structure or function persisted even after induction of pathological stress by Ang II. Ang II appeared to increase systolic blood pressure among control mice, but the lack of significance may reflect insufficient power due to sample size in our study. Nonetheless, Ang II significantly increased systolic blood pressure in TCDD-treated mice, as well as mean arterial pressure in TCDD-treated females, compared to controls, similar to findings previously reported^86^. Furthermore, using high-frequency echocardiography, we showed that Ang II increased LV mass normalized to body weight in females, and decreased stroke volume in males compared to saline controls. There was a trend towards Ang II-induced EDV among male mice, though this was not significant, also reflecting insufficient power to detect a difference. Finally, TCDD reduced cardiac fibrosis in both sexes, though this trend was only significant among mice receiving saline. Overall, the results of our findings related to Ang II effects suggest a sex-specific effect, but given the limited evidence for sexual dimorphism^87^, more research is needed in this area. Interestingly, continuous TCDD treatment from fertilization through adulthood reversed these effects in both sexes, and reduced cardiac fibrosis in male mice. In other studies involving *Ahr*^−/−^ mice, while *Ahr*^−/−^ mice developed severe cardiac fibrosis after Ang II infusion compared with wild-type mice^88^, these mice were also found to be hypotensive in response to Ang II, and *Ahr*^+/−^ mice were normotensive in response^89^. Furthermore, another study showed that treatment with tryptophan supplementation during pregnancy protects adult rat offspring from hypertension programmed by maternal chronic kidney disease^90^, suggesting possible dietary intervention therapies against pathologies related to the interplay between AHR and renin-angiotensin signaling. Taken together with our findings, the current research suggests that endogenous AHR signaling may play a role in developing ‘heart failure’ states, and that mitigation of this signaling such as via TCDD in adults may give rise to a ‘compensated’ state of mild hypertrophy in response to Ang II in comparison to a ‘heart failure’ state in corn oil controls. Building on our pilot study, future studies utilizing larger mouse sample sizes and across a range of time points are warranted. Specifically, time points for TCDD exposure from our study and those previously used in similar studies involving AHR and the renin-angiotensin system will need to validate our results and also address the context and temporally specific roles of AHR signaling in the development of hypertrophy and risk of heart failure in relation to the renin-angiotensin system. This can be also be performed by comparing *Ahr* ablation with activation by TCDD or endogenous ligands such as tryptophan during development and/or through adulthood.

## Conclusions

An array of interconnected compensatory responses are likely at play to mitigate the effects of developmental and continuous exposure to TCDD on heart development, structure, and function. Our findings also extended observations driven by TCDD that cardiovascular dysfunction related to exercise endurance was significantly more pronounced in males than in females by showing drastically lower performance in older mice compared to previous studies, underscoring the sexual dimorphism and impact of age in this phenotype. Finally, TCDD appeared to have blunting effects on myocardial hypertrophy. If these findings are confirmed using a larger study, they may provide insight into new AHR signaling pathways connected to the renin-angiotensin system that may be interesting therapeutic targets for the prevention of hypertrophy and the risk of HFpEF.

## Supplementary Information

Supplementary Information 1.

Supplementary Information 2.
