# Supplementary material for: Developmental and lifelong dioxin exposure induces measurable changes in cardiac structure and function in adulthood
Source: Sci Rep. 2021 May 17;11:10378. doi: 10.1038/s41598-021-89825-w (PMC8129097; doi:10.1038/s41598-021-89825-w)
Supplement: Supplementary file 1 — Supplementary Information 1. [file 41598_2021_89825_MOESM1_ESM.pdf]

Developmental and lifelong dioxin exposure induces measurable changes in cardiac structure and function in adulthood

Scientific Reports

Matthew de Gannes[1], Sheryl E. Koch[2], Alvaro Puga[1], and Jack Rubinstein[2*]

1 Department of Environmental and Public Health Sciences and Center for Environmental Genetics, University of Cincinnati College of Medicine, 160 Panzeca Way, Cincinnati, OH 45267, United States of America **2** Department of Internal Medicine, Division of Cardiovascular Health and Disease, University of Cincinnati College of Medicine, 3230 Eden Ave, Cincinnati, OH 45267, United States of America

* To whom correspondence should be addressed:
E-mail: rubinsjk@ucmail.uc.edu

Online Resource 1 - List of Contents

Supplemental Figures

a. Fig 1

b. Fig 2-3

c. Fig 4-6

Supplemental Tables

d. Table 1

e. Table 2

Supplemental Figures

Fig 1

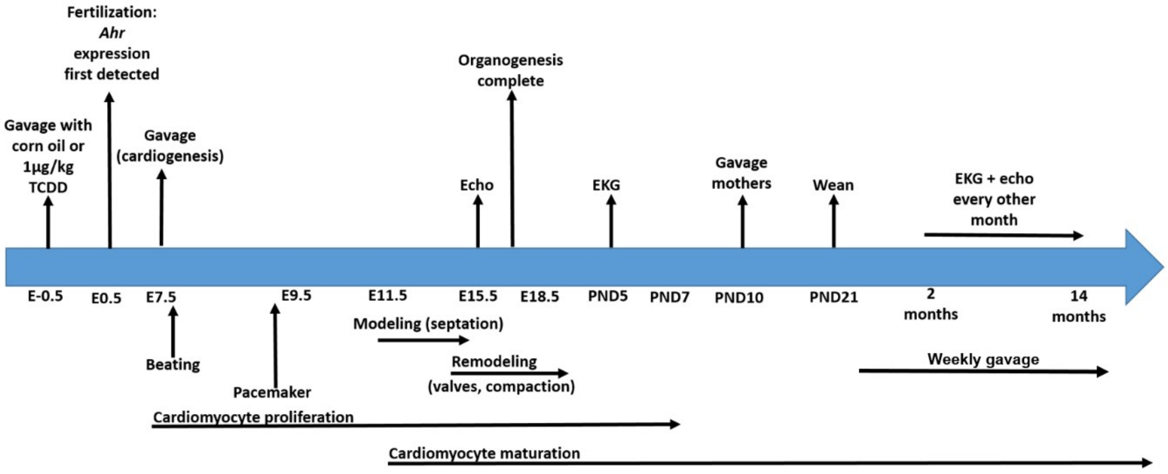

a. **Online Resource 1 Fig 1** - Experimental design for gestational exposure to the prototypical AHR ligand (TCDD) as well as echocardiography and electrocardiography studies.

Fig 2

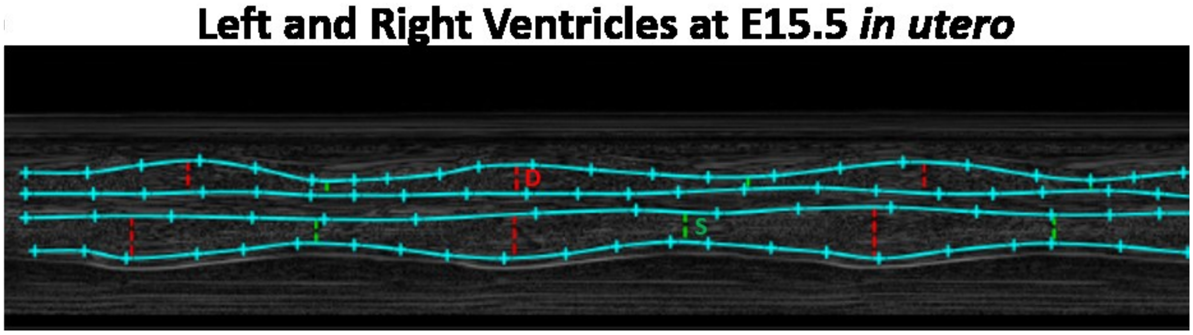

Fig 3

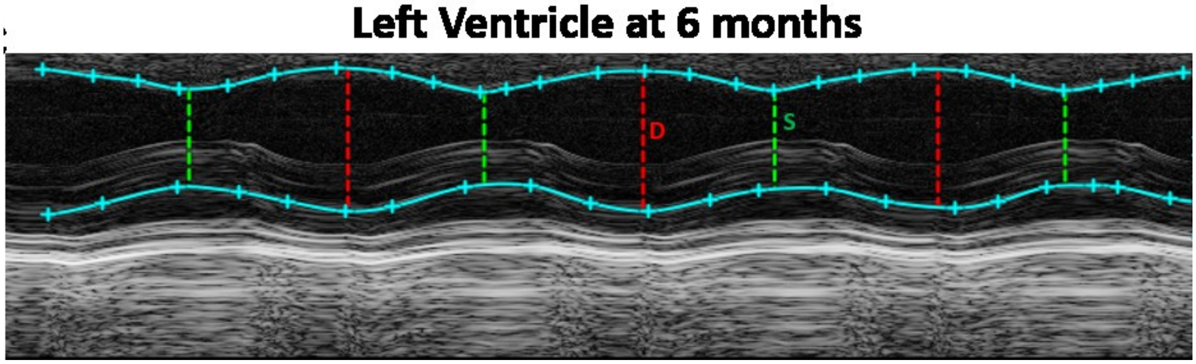

b. **Online Resource 1 Figs 2-3** - Annotated representative echocardiography illustrations in the left and right ventricles of an embryo *in utero* at E15.5 (2) and in the left ventricle at 6 months of age (3). *D* = diastole *S* = systole.

Fig 4

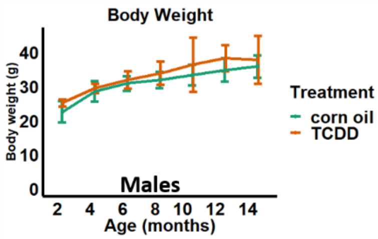

Fig 5

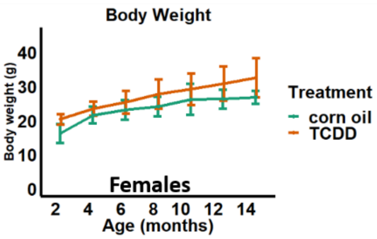

Fig 6

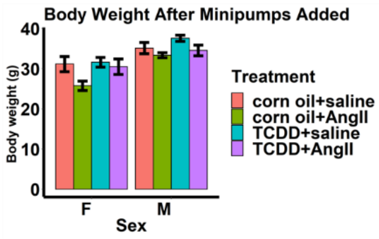

c. **Online Resource 1 Figs 4-6** - Body weight (g) from TCDD- and corn oil-treated males (4) and females (5) over the 14 month duration of the study and after surgical implantation of osmotic minipumps containing either Ang II or saline (6).

Supplemental Tables

d. **Online Resource 1 Table 1**- Mouse sample numbers over the 14 months of echocardiography and electrocardiogram studies

Month	2	4	6	8	10	12	14
Corn oil male	17	17	16	15	14	14	15
TCDD male	18	18	18	18	18	18	16
Corn oil female	18	18	13	13	14	12	12
TCDD female	14	14	14	14	14	14	14

e. **Online Resource 1 Table 2**- Treadmill exercise protocol mice followed on days 1 and 2 for acclimatization training, before the physical capacity test on day 3, and before endurance testing on day 4.

Protocol	Speed (m/min)	Time (minutes)	Shock Strength (mA)
Acclimatization	0	2	1
Warm-Up	10	5	1.5
Exercise	14	10	2
